# Data related to the mesoscopic structure of iso-graphite for nuclear applications

**DOI:** 10.1016/j.dib.2018.05.003

**Published:** 2018-05-09

**Authors:** Benjamin März, Kenny Jolley, Thomas James Marrow, Zhaoxia Zhou, Malcolm Heggie, Roger Smith, Houzheng Wu

**Affiliations:** a Department of Materials, Loughborough University, Leicestershire LE11 3TU, UK; b Department of Chemistry, Loughborough University, Leicestershire LE11 3TU, UK; c Department of Materials, University of Oxford, Parks Road, Oxford OX1 3PH, UK; d Department of Mathematical Science, Loughborough University, Leicestershire LE11 3TU, UK

## Abstract

The data in this article are related to the research article “Mesoscopic structure features in synthetic graphite” (März et al., 2018) [1]. Details of the manufacture of isostatically moulded graphite (iso-graphite), thin foil preparation by focused ion beams (FIB) for analysis, and characterisation methods are provided. The detailed structures of coke filler and binding carbon are presented through scanning electron microscopy (SEM), scanning transmission electron microscopy (STEM) and Raman spectroscopy characterisation. Atomistic modelling results of mesoscopic structural features are included.

**Specifications Table**

**Table:** 

Subject area	*Physics, materials science*
More specific subject area	*Structure of isotropic graphite for nuclear applications*
Type of data	*Images (electron microscopy), Figures (atomistic modelling), Text file*
How data was acquired	*SEM (Jeol JSM-7800F), STEM (JEOL JEM-ARM200F), FIB (FEI Nova 600 Nanolab)*
Data format	*Raw and analysed optical microscopy, SEM and STEM micrographs, Raman spectra as well as simulated images are presented.*
Experimental factors	*Machined samples of an as-manufactured isotropic graphite were broken for sampling and analysis. The fracture surfaces, that have coke fillers and binding carbon exposed, were analysed.*
Experimental features	*Raman spectroscopy was used to map the I*_*D*_*/I*_*G*_*Raman band ratio, to evaluate the differences of structural disorder, on the fracture surface. SEM imaging was applied to document structural features after fracture. Thin foil specimens were prepared by FIB from selected locations on the fracture surfaces. The structures were investigated at the atomic and mesoscopic levels. Raman spectroscopy measurements were correlated with the microscopic results. Atomistic modelling was applied to provide further insights into the atomistic structure of mesoscopic structural features in graphite.*
Data source location	*Loughborough University, Loughborough, United Kingdom (Latitude: 52.762000°, Longitude: -1.241000°)*
Data accessibility	*The experimental data and atomistic modelling results are available with this article.*
	*The Python code for generating the atomic structure is available in a public repository (K. Jolley,*https://github.com/Kenny-Jolley/Graphene*).*

**Value of the data**

• The mesoscopic structure of an unirradiated new isotropic graphite grade, SNG623, provides baseline information for further investigation of the same grade that is irradiated by neutrons or ions.
• A highlight of the manufacture of the graphite and representative properties of SNG623 is given for future reference.
• The sample preparation procedure of TEM thin foil specimens is provided as a guideline.
• Simulated images of atomistic structure are presented to provide further insights into the mesoscopic structures revealed by (S)TEM in isotropic graphite.
• Raman *I*_D_/*I*_G_ mapping data is given for correlating the measurements with mesoscopic structural features in graphite. This data is also provided for stimulating discussion about the potential of this technique, for quantitative studies of structural changes in graphite after irradiation, or manufacturing via different processing parameters or raw materials.

## 1 Data

### 1.1 Experimental data

**Fig. 1:**
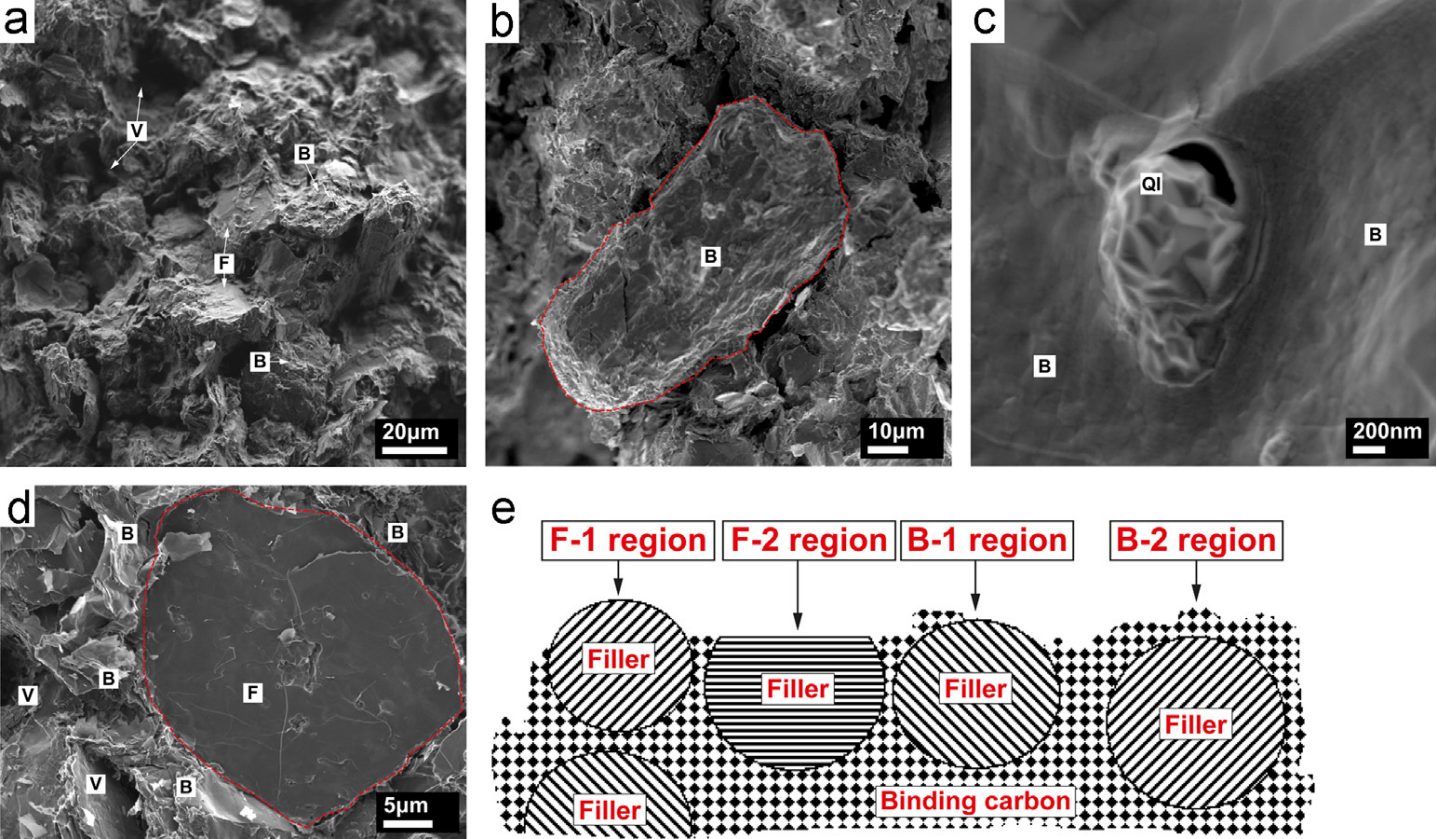
Morphologies of the as-fractured surface of isotropic graphite by snapping a sample. (a) overview of the surface, showing F, B and V regions with different morphologies; (b) detailed morphology in region B; (c) quinoline insoluble inclusions inside region B; (d) detailed morphology in region F; (e) a schematic cross section of the as-fractured surface showing F and B regions where F-1 represents exposed filler surface without binding carbon, F-2 filler subjected to trans-granular fracture, B-1 exposed filler with patched binding carbon attached on, and B-2 binding carbon only without filler exposed. Note, the voids are not shown in the schematic in (e).

Illustrated in Fig. 1(e) is a schematic of the microstructures found on a fracture surface of the investigated isotropic graphite. The given labels are used to distinguish between different regions found by scanning electron microscopy (SEM) imaging.

An overview of a fracture surface is shown in Fig. 1(a). There are characteristic regions that can be described as: flat and smooth, labelled “F”; rounded and rough, labelled “B”; and voids, labelled “V”. Most areas show the morphology labelled “B” and only a small number of regions have characteristics of those labelled “F”.

In Fig. 1(b) a single filler-like region, with typical features of region B, is outlined by a dashed red line along the interface with binding carbon. The topographic features of B vary in length scale from sub-micron up to a few microns. We believe that such features are formed by cracking, either through the binding carbon, or along an interface between binding carbon and filler. The features must then either mirror the structural units inside the binding carbon, or the surface topography of the filler coke. The binding carbon and filler have good coherency at some positions, but are detached at others. We may hence infer that, on the exposed surface of this filler, the viewed morphology is a mixture of a filler surface and a fracture surface of binding carbon. This mixed type of region, B-1, is schematically shown in Fig. 1(e). However, in most parts of the B region it is likely that only either binding carbon or filler is exposed; these are labelled B-2 or F-1 respectively, as schematically shown in Fig. 1(e). We judge that the regions in the top left corner and lower right corner in Fig. 1(b) are characteristic of the fracture surface of the binder, i.e. B-2.

Some spherical inclusion features, as shown in Fig. 1(c), are present in region B. These inclusions have the characteristic structure of the quinoline-insoluble (QI) particles, as reported by other researchers [2], [3], [4], [5], [6], [7]. They exist fairly extensively inside binding carbon, which is derived from coal tar pitch, but rarely inside the petroleum-derived coke. Through image analysis using ImageJ [8], a mean area fraction of ~10% QI was estimated on two TEM specimens, taken from two binding carbon regions. By taking 80 particles into account, the minimum and maximum circular diameters were 84.7 nm and 869.3 nm, respectively, with an average diameter of 259.0±166.7 nm.

Fig. 1(d) shows details in region F. The dimensions of this region (~17 μm×~14 μm) are close to the nominal diameter of coke filler (~20 μm), as specified by the manufacturer. Inside the dashed line, the surface is smooth and flat, consistent with the surface formed through cleaving parallel to the basal planes of graphite. Following the schematic in Fig. 1(e), this characteristic region is termed F-2. The material surrounding of this F region is judged to be binding carbon, as it shows the clear characteristics of region B-2.

**Fig. 2:**
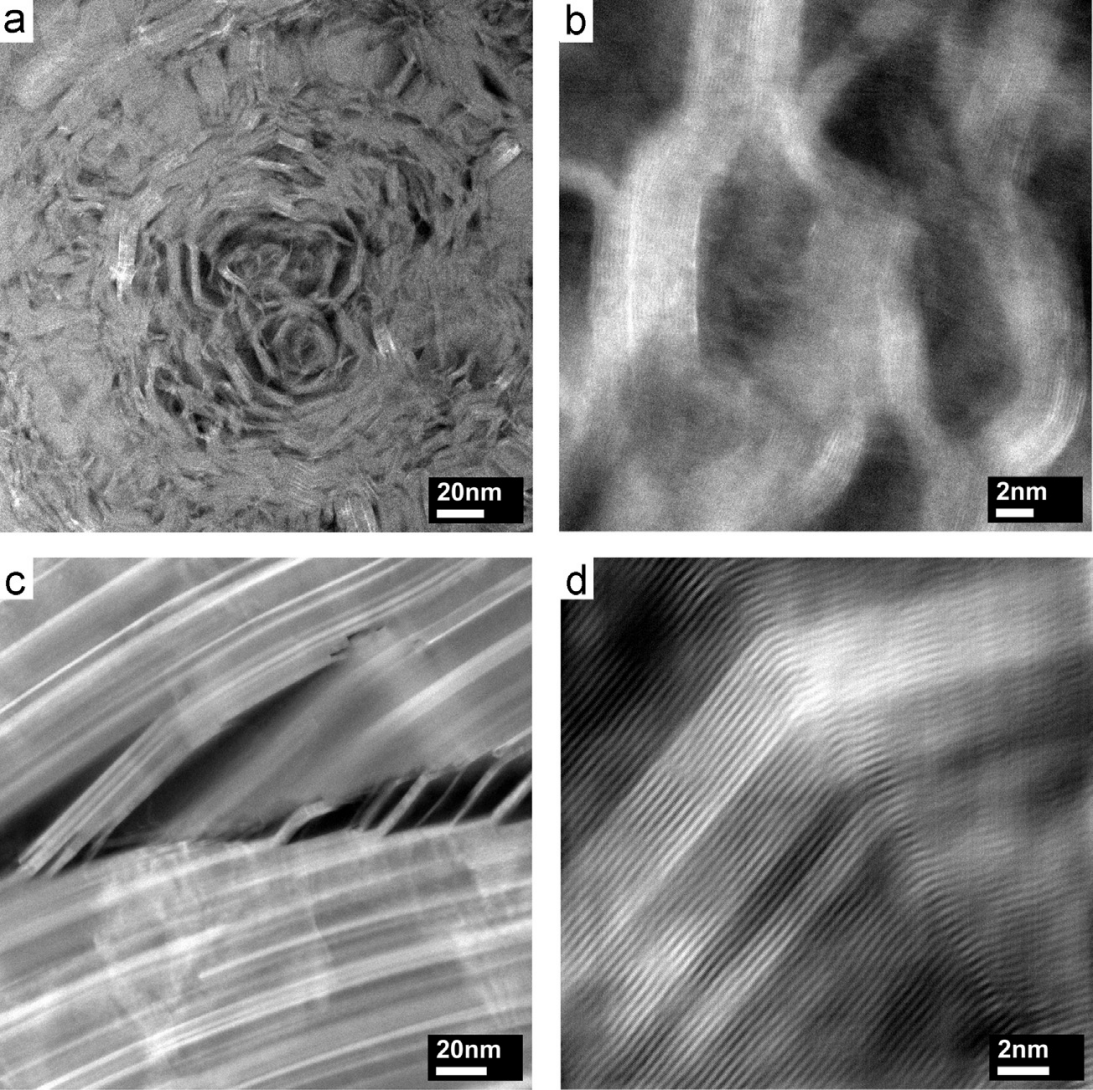
ADF-STEM images of QI particle (a) and corresponding detailed view of bent graphite slabs (b); graphite layers with delamination (c) and detailed view at a bend (d).

Fig. 2(a) shows an angular dark-field (ADF) scanning transmission electron microscopy (STEM) image of a spherical QI feature, found in the investigated graphite, measuring several hundred nanometres in diameter. These particles consist of slabs of well aligned graphite, measuring only around 5 nm in thickness. These slabs are delaminated and bent in different directions, as shown in Fig. 2(b), resulting in lengths of these perfect graphite domains of only 10–20 nm. Graphite exhibiting a more perfect structure of basal planes which are extended up to several 100 nm in length is shown in Fig. 2(c). In the centre of Fig. 2(c), delaminated graphite slabs can be seen, which are shown in more detail in Fig. 2(d). Contrast differences indicate the presence of twist boundaries between perfectly AB stacked graphite.

**Fig. 3:**
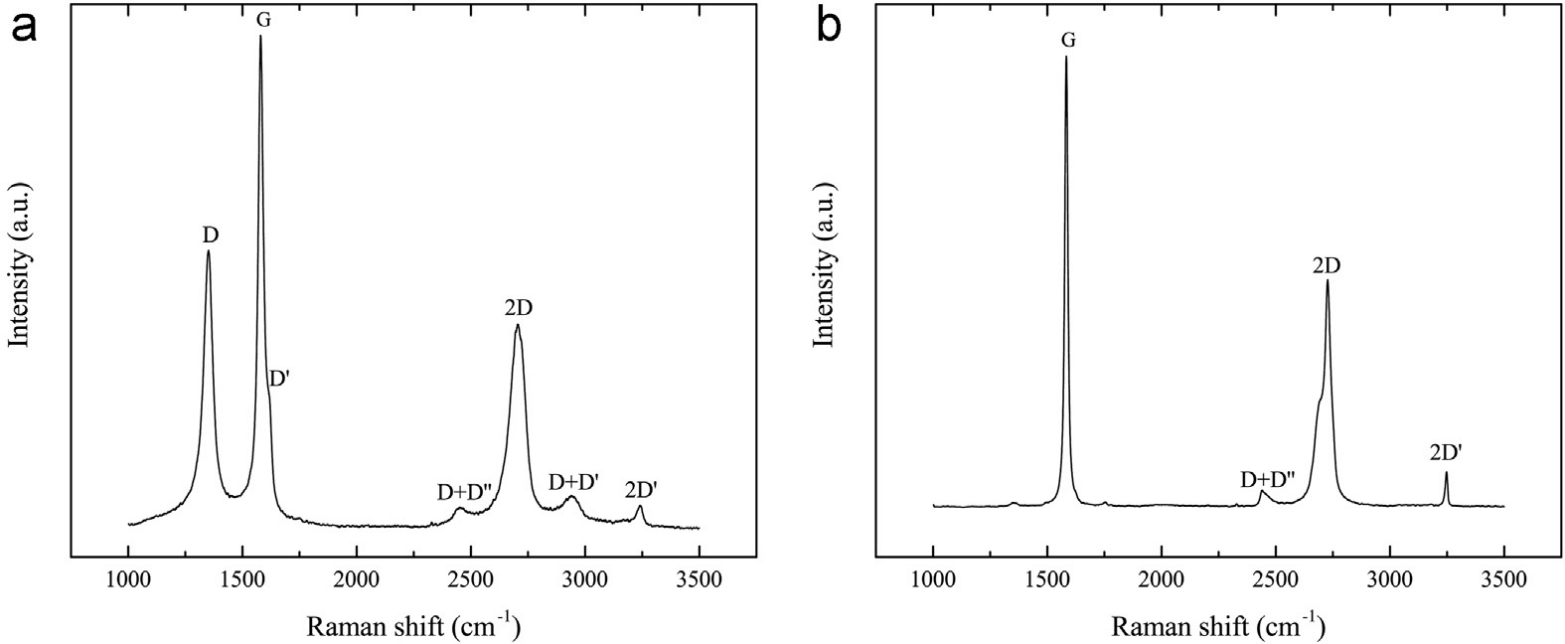
Representative Raman spectra of SNG623 (a) and HOPG (b), acquired using a 514 nm laser.

Fig. 3 shows representative Raman spectra of SNG623, acquired through randomly probing on an as-fractured surface, and of highly oriented pyrolytic graphite (HOPG). The most prominent features in the Raman spectrum of isotropic graphite are seen around 1350 cm^−1^, 1582 cm^−1^, and 1620 cm^−1^, known as the D, G and D′ band respectively [9]. The G band is characteristic of sp^2^ carbon networks, while the D and D′ bands are induced by a disordered structure and are not seen for a perfect graphite crystal [10]. The spectrum acquired of HOPG, which is almost perfect graphite, shows much weaker D and D′ bands than that of SNG623. The strong G band indicates that the *sp*^2^ bond is dominant in this graphite. In fact, *sp*^2^ might be the only type of bonding because no Raman bands around 1333 cm^−1^ and 1850–2100 cm^−1^, representing *sp*^3^ and *sp* bonds respectively [11], were observed.

**Fig. 4:**
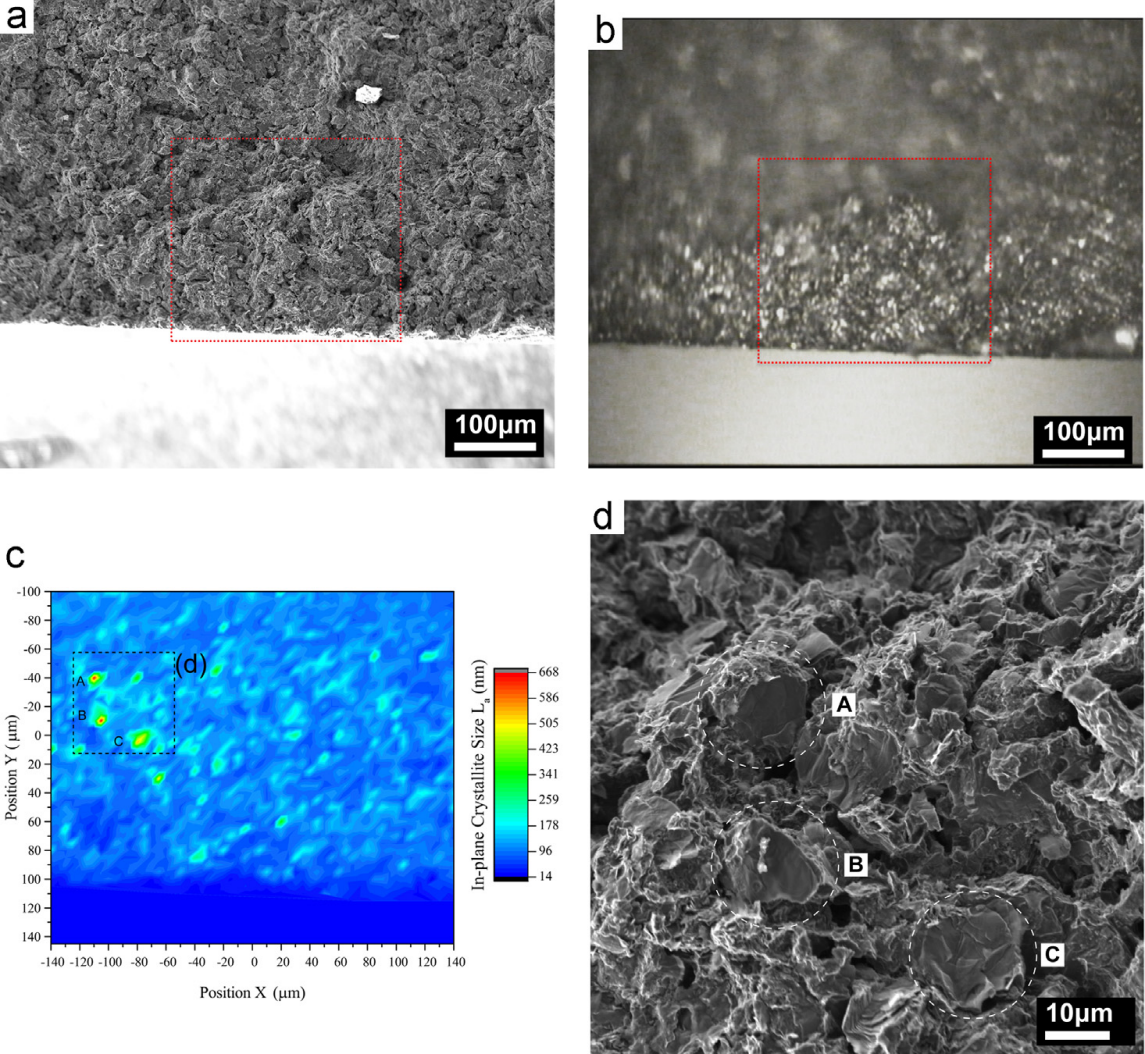
Raman data acquisition on a fracture surface of SNG623. SEM image of map location (a); light optical view of the location in the Raman microscope (b); in-plane crystallite size estimated from the *I*_D_/*I*_G_ ratio at each probed position (c); correlation of 3 selected locations showing a large *L*_a_ (low *I*_D_/*I*_G_) with the SEM image (d).

Fig. 4 shows the acquisition procedure of the Raman data which is used to distinguish different regions by their D band intensity, determined by the intensity ratio (ratio of fitted peak maxima) of D and G bands *I*_D_/*I*_G_. From each ratio, the in-plane crystallite size, *L*_a_, was estimated using the empirical equation given by Ref. [12].

Fig. 4(a) shows an overview taken by SEM of the analysed region, highlighted by a red square. The same region, viewed through the Raman microscope, is given in Fig. 4(b). The final map of the local distribution of *L*_a_ is presented in Fig. 4(c), showing localised peaks of high *L*_a_ at locations marked ‘A’, ‘B’ and ‘C’. Correlation with the SEM image shows, that at each of these locations, a cleaved flat graphite plane of a coke filler grain is exposed (Fig. 4(d)).

### 1.2 Mesoscopic structure modelling data

**Fig. 5:**
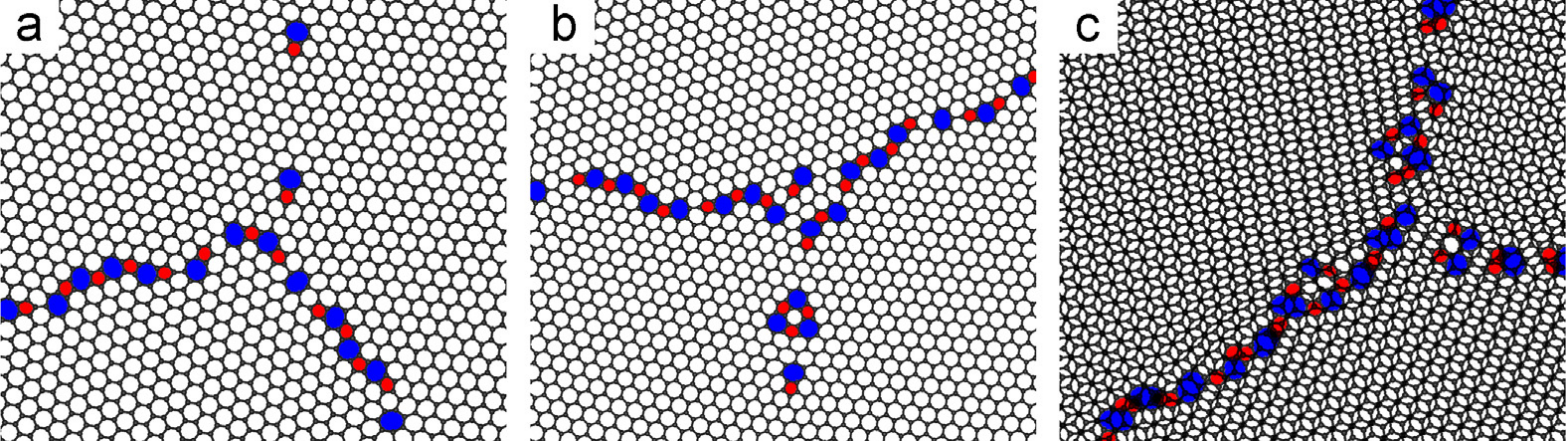
(a) An example of a triple junction at the apex of three graphite slabs. (b) Shows a different triple junction on the same surface. (c) Shows a triple junction with ‘A’ and ‘B’ graphite layers visible. The 7 member carbon rings are coloured blue and the 5 member carbon rings are coloured red.

Fig. 5 shows three examples of a triple junction that is composed of only 5, 6 and 7 carbon atom rings. The method for generating these structures is presented in the associated research article [1].

**Fig. 6:**
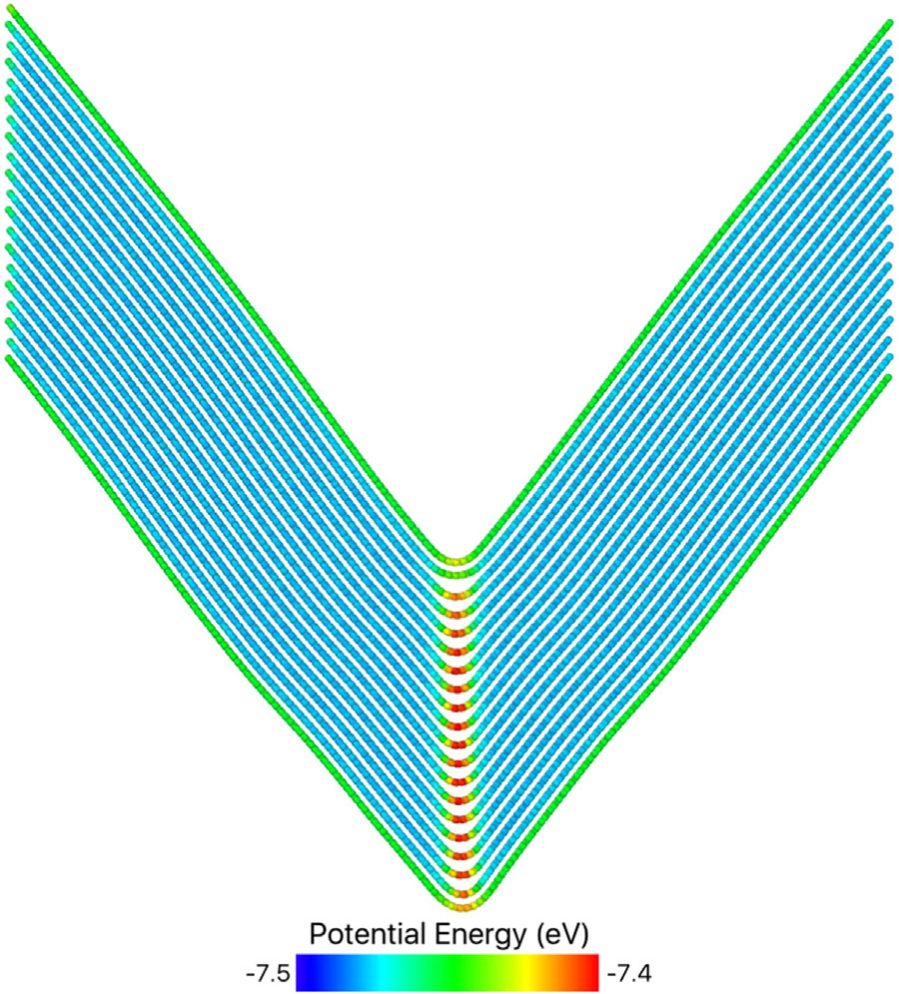
A graphite rod 100×4×10 unit cells (418×9.7×67 Å) bent in the centre. The angle between the graphite planes is 77.46°. This characteristic angle is the largest angle that maintains perfect AB stacking on each side, without basal defects. The structure shown has been minimised using the conjugate gradient algorithm. Each atom is coloured by its potential energy.

Sharp kink boundaries were also studied. An idealised sharp bend in the graphite planes was constructed by bending a rod of AB stacked graphite in the centre. The angle between the graphite planes was 77.46°. This is the largest angle which preserves the correct AB stacking on both sides, without any defects. The structure has fixed ends, periodic symmetry in the y direction, and free upper and lower surfaces. Fig. 6 shows the minimised structure after relaxation by the conjugate gradient method.

Molecular dynamics (MD) simulation of this structure was performed at 300 K for 100 ps to assess the stability of the structure. It was found that the structure appears stable, with only minor rounding of the sharp bend. This is in good agreement with the experimental observation of these kink boundaries. This idealised structure did not contain any defects, nor did any form during the MD simulation. All atoms remained in a 3-fold coordination state within 6 member hexagonal rings.

## 2 Materials and methods

Coke and coal tar pitch were used as raw materials of filler and binder of a moulded green body, respectively, for manufacturing this grade on a mass production line. Full details of the processing parameters are not disclosed due to commercial sensitivity, but in brief the average diameter of the filler particles in this grade is about 20 μm, and these were mixed with the pitch binder before isostatic pressing in a mould. The as-moulded green body was impregnated by pitch, followed by baking and graphitisation (>2800 °C). Representative properties of SNG623 were presented in the International Nuclear Graphite Specialist Meeting (INGSM-16) by the material supplier [13], and are summarised in Table 1.

**Table 1:** Representative properties of grade SNG623.

Density (g/cm^3^)	Young׳s modulus (GPa)	4-point flexural strength (MPa)	Compressive strength (MPa)	Electrical resistance (μΩ m)	Porosity (%)	CTE (20–600 °C) (10^−6^/°C)	Ash (ppm)
1.81	11	50	93	14	17	4.6	<10

In order to access the undamaged region of the samples, that have been previously machined by the manufacturer to tablets of 10 mm×0.5 mm in size, they were broken as follows: A graphite tablet was positioned over a small breaker bar to define the fracture location and a flat steel ruler was pressed on top of it with a bending force applied, until the sample broke. A broken piece was then glued using silver paint onto a specimen stub for further preparation.

TEM specimens were prepared using a focused ion beam (FIB) in a FEI Nova 600 Nanolab Dual Beam system. This method allowed the preparation of a TEM specimen from a position of particular interest on the fracture surface, e.g. from a selected coke filler or binding carbon.

**Fig. 7:**
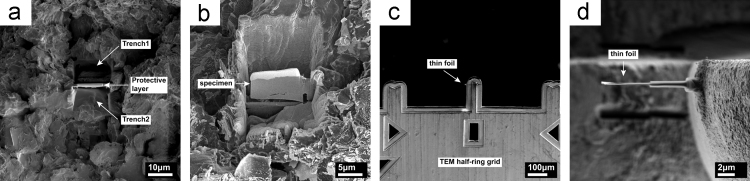
Shows the thin foil preparation procedure. Two trenches adjacent to the Pt protective layer (a); tilted sample showing the cut off specimen (b); specimen already transferred and welded to TEM Cu grid (c); top view of thinned specimen on the grid, showing slight bending due to low thickness (d).

A platinum coating of 1.5 μm thickness was deposited on the region of interest to prevent Ga^+^ implantation, followed by producing rectangular trenches adjacent to the platinum strip using a 20 nA Ga^+^ ion beam at 30 kV (Fig. 7(a)). After the thickness of the wall was reduced to ~5 μm, both sides were milled using 7 nA, followed by 3 nA Ga^+^ beams at 30 kV to clean and smooth the surfaces of the cross section to a thickness of 1.5 μm. Fig. 7(b) shows the TEM specimen after it was cut from the bulk using the ion beam. The TEM specimen was lifted out using a micro manipulator and welded onto a Cu half-ring grid, as shown in Fig. 7(c). A final thinning process was carried out using a 1 nA Ga^+^ beam down to 100 pA at 30 kV at a glancing incidence of 1.5° on both sides of the thin foil, to achieve the final thinning and to minimise the depth of any residual damage by the Ga^+^ ion beam, if any (Fig. 7(d)). Finally, a low-voltage polishing step using 70 pA Ga^+^ ions at 5 kV was applied at an angle of 4° to both surfaces of the foil to reduce the amorphous layer thickness.

High resolution annular dark-field (ADF) scanning transmission electron microscopy (STEM) was carried out using an aberration corrected (Cs) JEOL JEM-ARM200F microscope, operated at 80 kV.

The acquisition of Raman spectra was conducted using a Horiba Jobin-Yvon LabRam HR high spectral resolution Raman system with an integral confocal microscope. A laser wavelength of 514 nm (green) was used. Frequency calibration was done using the 520.7 cm^−1^ peak of a silicon wafer.

Using an integration time of 5 s, two spectra were accumulated at each position. A spectral range of 1200–1675 cm^−1^ and a diffraction grating of 1800 grooves/mm was used. The spectra were processed by first applying a spike removal filter, followed by a zero shift, then normalised and de-noised. For spectrum analysis, a Lorentzian function was fitted to each peak, and the peak intensities at the D- and G band positions, *I*_D_ and *I*_G_ respectively, were taken from the Lorentzian function heights.

## References

[1] März B., Jolley K., Marrow T.J., Zhou Z., Heggie M., Smith R., Wu H. (2018). Mesoscopic structure features in synthetic graphite. Mater. Des..

[2] Wen K.Y., Marrow T.J., Marsden B.J. (2008). The microstructure of nuclear graphite binders. Carbon.

[3] Karthik C., Kane J., Butt D.P., Windes W.E., Ubic R. (2012). Microstructural characterization of next generation nuclear graphites. Microsc. Microanal..

[4] Freeman H.M., Jones A.N., Ward M.B., Hage F.S., Tzelepi N., Ramasse Q.M., Scott A.J., Brydson R.M.D. (2016). On the nature of cracks and voids in nuclear graphite. Carbon.

[5] Krishna R., Jones A.N., Edge R., Marsden B.J. (2015). Residual stress measurements in polycrystalline graphite with micro-Raman spectroscopy. Radiat. Phys. Chem..

[6] Krishna R., Jones A.N., Marsden B.J. (2015). Transmission electron microscopy, Raman and X-ray photoelectron spectroscopy studies on neutron irradiated polycrystalline graphite. Radiat. Phys. Chem..

[7] Zheng G., Xu P., Sridharan K., Allen T. (2014). Characterization of structural defects in nuclear graphite IG-110 and NBG-18. J. Nucl. Mater..

[8] Schneider C.A., Rasband W.S., Eliceiri K.W. (2012). NIH Image to ImageJ: 25 years of image analysis. Nat. Methods.

[9] Tuinstra F., Koenig J.L. (1970). Raman spectrum of graphite. J. Chem. Phys..

[10] Lasithiotakis M., Marsden B.J., James Marrow T. (2013). Annealing of ion irradiation damage in nuclear graphite. J. Nucl. Mater..

[11] Pimenta M.A., Dresselhaus G., Dresselhaus M.S., Cançado L.G., Jorio A., Saito R. (2007). Studying disorder in graphite-based systems by Raman spectroscopy. Phys. Chem. Chem. Phys..

[12] Cançado L.G., Takai K., Enoki T., Endo M., Kim Y.A., Mizusaki H., Jorio A., Coelho L.N., Magalhães-Paniago R., Pimenta M.A. (2006). General equation for the determination of the crystallite size La of nanographite by Raman spectroscopy. Appl. Phys. Lett..

[13] H. Yang, H. Li, D. Huang, H. Wu, M. Snead, A.A. Campbell, Y. Katoh, T.D. Burchell, Specimen Size Effect on Type-I Fracture Toughness (K_IC_) Measurements of Fine Grain Nuclear Grade Graphite, in: Proceedings of the International Nucl. Graph. Spec. Meet. INGSM-16, Nottingham, 2015.

