# Supplementary material for: Data related to the mesoscopic structure of iso-graphite for nuclear applications
Source: Data Brief. 2018 May 9;19:651–9. doi: 10.1016/j.dib.2018.05.003 (PMC5997625; doi:10.1016/j.dib.2018.05.003)
Supplement: Supplementary file 1 — Supplementary material [file mmc1.docx]

# Declare of Interest

For this submission, declarations of interest: none
